# Polyhexanide and hydrogen peroxide inhibit proteoglycan synthesis of human chondrocytes

**DOI:** 10.1179/014788811X12949268296121

**Published:** 2011-03

**Authors:** Eric Röhner, Paula Hoff, Tobias Winkler, Philipp von Roth, Jörn Bengt Seeger, Carsten Perka, Georg Matziolis

**Affiliations:** 1 Department of Traumatology and Orthopaedics; 2 Department of Rheumatology and Clinical Immunology, Charité - Universitätsmedizin, Berlin, Germany

**Keywords:** Antiseptics, Human chondrocytes, Hydrogen peroxide, Polyhexanide, Proteoglycans

## Abstract

The use of local antiseptics is a common method in septic joint surgery. We tested polyhexanide and hydrogen peroxide, two of the most frequently used antiseptics with high efficacy and low toxicity. The purpose of this study was to evaluate the effects of both antiseptics on the extracellular cartilaginous matrix synthesis of human chondrocytes. Chondrocytes were isolated from donated human knee joints, embedded in alginate beads, and incubated for 10 and 30 minutes with polyhexanide (0.04%), hydrogen peroxide (3%), or phosphate-buffered saline (PBS) for control. Cartilaginous matrix production was quantified through light microscopic analysis of Alcian blue staining. Cell number and morphology were detected by histological analysis. Chondrocytes showed a decreased intensity of blue colouring after antiseptic treatment versus PBS. In contrast to that, neither the cell number per view field nor the cell morphology differed between the groups. Polyhexanide has more toxic potential than hydrogen peroxide. Based on the fact that the cell number and morphology was not altered by the substances at the examined concentrations, the lower intensity of Alcian blue staining of treated chondrocytes indicates a decreased cartilage-specific matrix synthesis by polyhexanide more than by hydrogen peroxide and control.

## Introduction

Different studies showed that shoulder and knee joint infections often result from infiltrations with local anaesthetics, glucocorticoids, or hyaluronic acid contrary to hip joint infections which are rarely infiltrated for diagnostic or therapeutic reasons.^1^–^3^ In addition to arthroscopic or open debridement, the application of local antiseptics is a standard procedure in the treatment of joint infections. The most frequently used antiseptics of local joint and wound infections are polyhexanide, and hydrogen peroxide. In contrast to antibiotic substances, they do not differ between procariontic and eukaryontic cells. Therefore, the tissue toxicity of antiseptic substances has to be determined for every tissue coming into contact. There is an ongoing discussion between elimination of bacteria and toxicity against chondrocytes.^4^,^5^ It should be assumed that the time of chondrocytes contact with local antiseptics should be between 10 and 30 minutes to eliminate most colonies of bacteria and though to limit chondrocyte toxicity. The most frequent bacteria, which are responsible for wound and joint infections, are staphylococci, streptococci, and Gram-negative bacteria.^1^,^6^–^8^ Besides inducing cell death, antiseptic substances may alter the cartilage-specific synthesis of proteoglycan in chondrocytes and thereby affect the cartilage tissue. The undisturbed interaction between the chondrocytes and extracellular matrix plays an important role for the regulation of chondrocyte homeostasis, which is important for the maintenance, the development, and the stabilisation of chondrocyte phenotype.^9^–^12^ Consequently, an interruption of proteoglycan synthesis results in cartilage damage with the loss of chondrocytes.^9^,^13^

Two of the most commonly used antiseptics in septic joint surgery are polyhexanide and hydrogen peroxide. Literature reviews show insufficient data about the effects of antiseptics on human cartilage. Two studies, which detected effects of antiseptics on bovine chondrocytes, were by Bates and co-workers and Müller and colleagues.^14^,^15^ In the study of Bates and colleagues, bovine articular cartilage was treated with hydrogen peroxide and showed a significant inhibition of proteoglycan synthesis. This result indicates that hydrogen peroxide is an active species of oxygen-derived reactive species, which is involved in the inhibition process.^15^ In the study of Müller and Kramer, articular cartilage of bovine sesamoid bone was treated with betaisodona, polyhexanide, and octenidine in the presence of *Escherichia coli* and *Staphylococcus aureus*. One result of this study was that all tested antimicrobial irrigation fluids inhibit proteoglycan synthesis. Additionally, Müller and Kramer could show that betaisodona has the capacity to destroy microorganism *in vitro* in the presence of cartilage tissue without negative impact on chondrocyte metabolism.^14^ In both studies, the mechanisms by which antiseptics cause an inhibition of proteoglycan synthesis in cultured articular cartilage were not clear. In the present study, we evaluate the effects of polyhexanide and hydrogen peroxide on the extracellular cartilaginous matrix synthesis of primary human chondrocytes.

## Materials and Methods

Tissue culture plasticware were obtained from TPP (Trasadingen, Switzerland). Culture medium, phosphate-buffered saline (PBS), trypsin, and fetal calf serum were purchased from Biochrom (Berlin, Germany). All other reagents were obtained from Sigma-Aldrich (Deisenhofen, Germany).

### Chondrocyte isolation and three-dimensional culture in alginate beads

Chondrocyte isolation was performed as previously described.^16^,^17^ Cartilage was obtained from four human donors with knee osteoarthritis not presenting any kind of infectious signals. Experimental protocols were approved by the local ethics committee. Cartilage was minced and digested in medium containing 1 mg/ml pronase (Sigma-Aldrich, Deisenhofen, Germany) for 30 minutes at 37°C. Next, digestion medium was discarded and the tissue was digested with medium containing 1 mg/ml clostridial collagenase (Sigma-Aldrich) for 18 hours at 37°C. Digested solution was filtered (70 μm Nylon; BD Falcon, Bedford, Germany) and centrifuged at 1200 rev/min for 8 minutes. The supernatant was discarded and the cell pellet was washed three times with PBS. Then chondrocytes were suspended in DMEM/Ham’s F12 with 10% fetal bovine serum, 1% penicillin/streptomycine, and cultured at 37°C, 95% air and 5% CO_2_ before use (Fig. 4). After confluence, chondrocytes were detached with 2 ml trypsin EDTA solution, and were counted by using Casy Cell-Counter. Cells numbering 3×10^6^ was performed with 600 ml of 4.8% alginate gel (240 mg alginate powder with 5 ml NaCl). For production of the alginate beads, an 18-gauge needle and a 2 ml syringe were used. Each bead had the same volume of 0.1 ml with approximately 5×10^5^ cells and was dropped from a height of 10 cm into 10 ml of non-stirred calcium chloride solution with a molarity of 0.102. Then alginate beads were hardened for 10 minutes and were suspended in six-well plates with 3 ml chondrogenic medium (DMEM/Ham’s F12 with 10% fetal bovine serum, 1% penicillin/streptomycine, 2% glutamine sulphate, ascorbic acid, TGF-beta, and dexamethasone) per well and cultured at 37°C, 95% air and 5% CO_2_ for 3 days before use.

### Treatment of alginate beads

Alginate beads with chondrocytes were cultured in six-well plates with 3 ml of chondrogenic medium. Two times (10 and 30 minutes), three antiseptic treatments (polyhexanide, hydrogen peroxide, or PBS control), and four replicates for each condition were assessed. After removal of the medium, 3 ml of 100% of concentrated 0.04% polyhexanide (Charité, Berlin, Germany) or 3% hydrogen peroxide (Charité), or PBS (control) were added for 10 and 30 minutes. After treatment with antiseptics, all alginate beads were washed three times with PBS and were re-suspended in chondrogenic medium for 3 weeks.

### Alcian blue staining

After 3 weeks of cultivation in chondrogenic medium at 37°C, 95% air, and 5% CO_2_, alginate beads were washed twice with PBS, fixed with 4% formalin, and rinsed again for 30 minutes. After cell staining with haematoxylin and eosin (Chroma, Brattleboro, VT, USA), the fixed alginate beads were dehydrated with a rising series of graded ethanol (10 minutes with 70%, 25 minutes with 80%, 25 minutes with 90%, 30 minutes with 96%, 1 hour with 100%) and soaked in xylene for 30 minutes. Then alginate beads were infiltrated with 60°C paraffin for 60 minutes and were sectioned into 4 μm sections. Sections were floated in a 56°C distilled water bath and collected on slides. These slides were soaked in xylene for 10 minutes and dipped in a declining series of graded ethanol (2 minutes in 100%, 2 minutes in 96%, 2 minutes in 90%, 2 minutes in 80%, 2 minutes in 70%, and 2 minutes in distilled water). Next, cleaned sections were irrigated with 3% acetic acid for 3 minutes and were stained with 1% Alcian blue in 3% acetic acid (pH 2.5) for 30 minutes. After a short irrigation with 3% acetic acid (pH 2.5) and distilled water for three times, the slides were coverslipped with Vitro-Clud-coverslipping medium. With the high concentration of proteoglycans that are characteristic for extracellular cartilaginous matrix, the sections acquired an intensive blue colouring. Light microscopy analysis of sections was performed using an Axiovert 40C light microscope (Zeiss, Göttingen, Germany). The view fields were digitized using a digital camera (Canon EOS 500D, 15.1 Megapixels), and the photographs were analysed using ImageJ (GNU License) by manually defining regions of interest in representative cell/matrix areas and areas without matrix or cells for blank. The blue level of colour histograms (8 bit for blue, red, and green) of both regions was measured. The matrix proteoglycan concentration was assumed to be positively correlated with the blue level value, i.e. a higher blue value represents a higher proteoglycan concentration.

### Detection of cell number and morphological characteristics

Chondrocytes embedded in alginate beads were incubated with polyhexanide or hydrogen peroxide, sectioned into 4-μm sections, and stained with 1% Alcian blue. PBS-treated chondrocytes were used as control. Analysis of the plates was performed by using Axiovert 40C light microscope (Zeiss). Cell number was detected by counting cells per view field (lens: 10×0.25, ocular: 10×18).

### Statistical analysis

Statistical tests were performed using Graph Pad Prism Software (Graph Pad Software, San Diego, CA, USA). Data were expressed as mean±standard deviation of the mean (SEM). Statistical differences were verified using a non-parametric Wilcoxon matched-pairs test as indicated in the legends. A *P*-value of <0.05 was considered significant.

## Results

**Figure 1:**
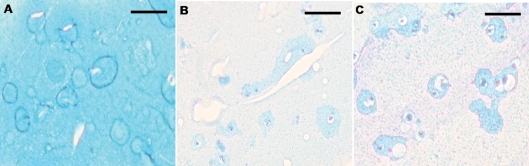
Alcian blue staining of chondrocyte after exposure to antiseptics. Chondrocytes were treated for 30 minutes with PBS, polyhexanide, or hydrogen peroxide, and were analysed by light microscopy. (A) PBS-treated control chondrocytes; (B) chondrocytes incubated with polyhexanide; (C) chondrocytes treated with hydrogen peroxide. One representative is shown from at least three independently performed experiments.

**Figure 2:**
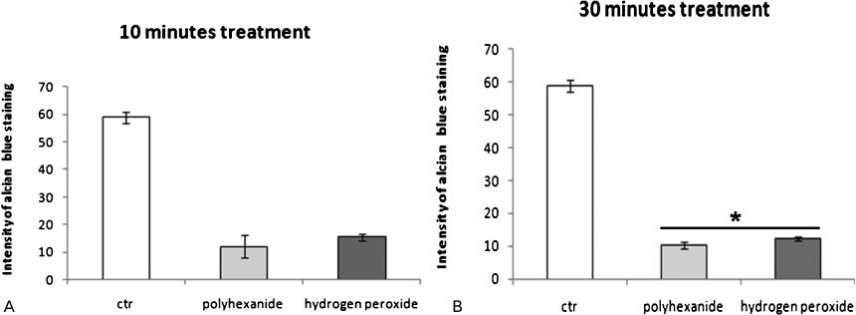
Intensity of Alcian blue after treatment with polyexanide or hydrogen peroxide. Chondrocytes treated with antiseptics were compared with PBS-treated chondrocytes (control) after (A) 10 and (B) 30 minutes. *n* = 4, mean±SEM, non-parametric Wilcoxon matched-pairs test. As compared to CTR, *P<0.05.

Histological analysis by Alcian blue staining showed a significantly decreased intensity of blue coloring after antiseptics treatment for 10 and 30 minutes versus the control group (Figs. 1 and 2). The positive Alcian blue staining of the control group indicates a high concentration of proteoglycans (58.92±1.96). In contrast, a decrease of Alcian blue staining suggests a lower concentration of proteoglycans seen in chondrocytes incubated in the indicated antiseptics (Figs. 1 and 2).

**Figure 3:**
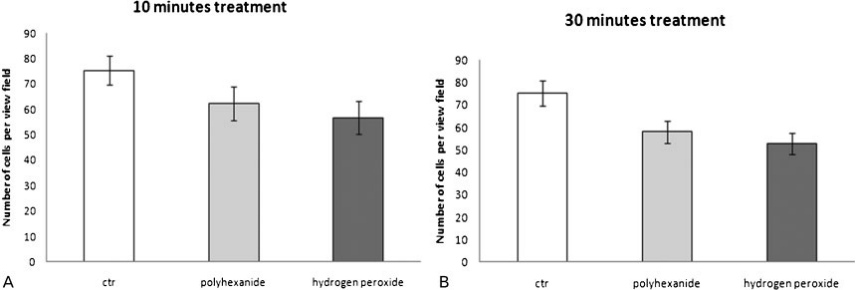
Cell number of chondrocytes per view field after treatment with PBS, polyhexanide, or hydrogen peroxide. Chondrocytes treated with antiseptics were compared with PBS-treated chondrocytes (control) after (A) 10 and (B) 30 minutes. *n* = 4, mean±SEM.

The comparison between polyhexanide- and hydrogen peroxide-incubated chondrocytes shows that human chondrocytes have a higher intensity of Alcian blue staining after treatment with hydrogen peroxide than after treatment with polyhexanide. This was detected for each treatment time point (10 minutes: 15.49±1.29 versus 12.11±4.13; 30 minutes: 12.31±0.56 versus 10.27±1.03; *P*<0.05) (Fig. 2). Analysis of the cell number per view field and morphological characteristics showed no significant differences between control (PBS), and between hydrogen peroxide or polyhexanide (Figs. 2 and 3).[Fig his-34-01-035-f04]

**Figure 4 his-34-01-035-f04:**
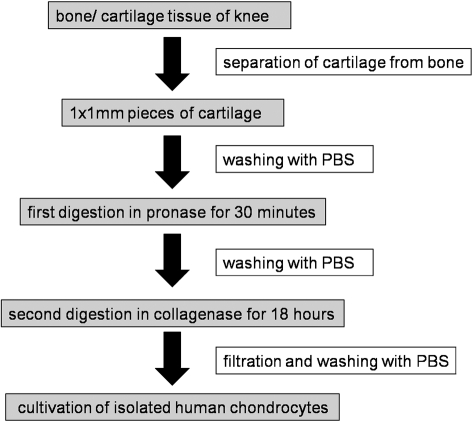
Experimental protocol for chondrocyte isolation from cartilage tissue.

## Discussion

The optimal therapy of joint infections is currently still an unsolved problem.^18^ In contrast to treatment using antibiotics which has brought a therapeutic window based on different mechanisms of action, antiseptics act through their more or less undifferentiated cell toxicity. Despite their importance in eradication of bacteria, the damage of antiseptic application to cartilage tissue is still investigated insufficiently.^19^ Our results show that both polyhexanide and hydrogen peroxide inhibit proteoglycan synthesis of human chondrocytes, which could possibly result in cartilage degradation. Additionally, we could show that polyhexanide inhibits proteoglycan synthesis more than hydrogen peroxide. Several studies indicated that a dysbalance in chondrocyte homeostasis, induced by antiseptics or antibiotics, results in a degradation of cartilage tissue.^14^,^15^,^19^ Taking other studies into account, the aim of the present study was to compare the effects of polyhexanide and hydrogen peroxide, representing two of the most frequently used antiseptics, on the extracellular cartilaginous matrix synthesis of human chondrocytes. Resulting from the fact that in monolayer culture the diffusion barrier is minimal, *in vitro* results show higher cell toxicity than *in vivo* examinations. A chondrocyte culture with a stable phenotype and a cartilage-like barrier for antiseptics influence was achieved by using an alginate matrix.^12^ The incubation times (10 and 30 minutes) represent a clinically relevant dimension in septic joint surgery and should therefore allow conclusions from the *in vitro* data.

In addition to our analysis of cartilaginous matrix production, we detected the cell number per view field and cell morphology of the treated articular chondrocytes. Unexpectedly, the cell number and morphology did not significantly differ between control group and antiseptic treated chondrocytes embedded in alginate beads. Contrary results were shown by a study of Ince and colleagues or Charalambous and co-workers.^2^,^19^ In the study of Ince and colleagues, human osteoblasts and endothelial cell were incubated with different concentrations of polyhexanide for 6 hours. Already a low concentration of 0.0006% of polyhexanide demonstrated a significant decrease of total cell number and viability.^19^ In the study of Schaumburger and co-workers, cultured chondrocytes were treated with different concentrations of polyhexanide, hydrogen peroxide, and betaisodona. In all treatment groups, a decrease of vital cells and of total cell count were detected.^5^ In comparison with the results of the present study, our data showed no significant differences of cell numbers between control and antiseptic-treated chondrocytes. An explanation is very likely that chondrocytes embedded in alginate beads show a higher and clinically more relevant barrier against the injurious effects of antiseptic solutions. Monolayer-cultured chondrocytes do not have any barrier against toxic solutions.

In summary, polyhexanide has more toxic potential than hydrogen peroxide. Both antiseptics inhibit the proteoglycan synthesis of human chondrocytes. Based on the fact that the cell number and morphology were not altered by the substances at the examined concentrations, the lower intensity of Alcian blue staining of treated chondrocytes indicates a direct decrease of cartilage specific matrix synthesis by polyhexanide more than by hydrogen peroxide and control.

## References

[1] Armstrong RW, Bolding F, Joseph R (1992). Septic arthritis following arthroscopy: clinical syndromes and analysis of risk factors. Arthroscopy.

[2] Charalambous CP, Tryfonidis M, Sadiq S, Hirst P, Paul A (2003). Septic arthritis following intra-articular steroid injection of the knee — a survey of current practice regarding antiseptic technique used during intra-articular steroid injection of the knee. Clin Rheumatol.

[3] von Essen R, Savolainen HA (1989). Bacterial infection following intra-articular injection — a brief review.. Scan J Rheumatol.

[4] Kalteis T, Lüring C, Schaumburger J, Perlicl L, Bäthis H, Grifka J (2003). Gewebetoxizität lokaler Antiseptika.. Z Orthop.

[5] Schaumburger J, Beckmann J, Springorum HR, Handel M, Anders S, Kalteis T (2010). Toxitität lokaler Antiseptika auf Chondrozyten *in vitro*. Z Orthop Unfall.

[6] Riegels-Nielsen P, Frimodt-Möller N, Jensen J (1987). Rabbit model of septic arthritis. Acta Orthop Scand.

[7] Ryan MJ, Kavanagh R, Wall PG, Hazleman BL (1997). Bacterial joint infection in England and Wales: analysis of bacterial isolates over a four year period. Br J Rheumatol.

[8] Smith M (1986). Arthroscopic treatment of the septic knee. Arhtroscopy.

[9] Muir H (1995). The chondrocyte, architect of cartilage. Biomechanics, structure, function and molecular biology of cartilage matrix macromolecules. Bioessays.

[10] Goldring MB (2000). The role of the chondrocytes in osteoarthritis. Arthritis Rheum.

[11] Newman AP (1998). Articular cartilage repair. Am J Sports Med.

[12] de Crombrugghe B, Lefebvre V, Behringer RR, Bi W, Murakami S, Huang W (2000). Transcriptional mechanisms of chondrocytes differentiation. Matrix Biol.

[13] Goldring MB (2006). Update on the biology of the chondrocyte and new approaches to treating cartilage disease. Best Pract Res Clin Rheumatol.

[14] Müller G, Kramer A (2005). Effect of selected wound antiseptics on adult articular cartilage (bovine sesamoid bone) in the presence of *Escherichia coli* and *Staphylococcus aureus*. J Orth Res.

[15] Bates EJ, Johnson CC, Lowther DA (1984). Inhibition of proteoglycan synthesis by hydrogen peroxide in cultured bovine articular cartilage. Biochimica Biophysica Acta.

[16] Röhner E, Detert J, Kolar P, Hocke A, N’Guessan P, Matziolis G (2010). Induced apoptosis of chondrocytes by *Porphyromonas gingivalis* as a possible pathway for cartilage loss in rheumatoid arthritis. Cal Tiss Int.

[17] Pischon N, Röhner E, Hocke A, N’Guessan P, Müller HC, Matziolis G (2009). Effects of *Porphyromonas gingivalis* on cell cycle progression and apoptosis of primary human chondrocytes. Ann Rheum Dis.

[18] Stutz G, Gächter A (2001). Diagnostik und stadiengerechte Therapie von Gelenkinfekten. Unfallchirurg.

[19] Ince A, Schütze N, Hendrich C, Jakob F, Eulert J, Löhr JF (2007). Effect of polyhexanide and gentamicin on human osteoblasts and endothelial cells. Swiss Med Wkly.

